# Mathematical Modeling of Intestinal Iron Absorption Using Genetic Programming

**DOI:** 10.1371/journal.pone.0169601

**Published:** 2017-01-10

**Authors:** Andrea Colins, Ziomara P. Gerdtzen, Marco T. Nuñez, J. Cristian Salgado

**Affiliations:** 1 Laboratory of Process Modeling and Distributed Computing, Department of Chemical Engineering and Biotechnology, University of Chile, Santiago, Chile; 2 Centre for Biotechnology and Bioengineering, Department of Chemical Engineering and Biotechnology, University of Chile, Santiago, Chile; 3 Iron and Biology of Aging Laboratory, Department of Biology, Faculty of Sciences, University of Chile, Santiago, Chile; CINVESTAV-IPN, MEXICO

## Abstract

Iron is a trace metal, key for the development of living organisms. Its absorption process is complex and highly regulated at the transcriptional, translational and systemic levels. Recently, the internalization of the DMT1 transporter has been proposed as an additional regulatory mechanism at the intestinal level, associated to the mucosal block phenomenon. The short-term effect of iron exposure in apical uptake and initial absorption rates was studied in Caco-2 cells at different apical iron concentrations, using both an experimental approach and a mathematical modeling framework. This is the first report of short-term studies for this system. A non-linear behavior in the apical uptake dynamics was observed, which does not follow the classic saturation dynamics of traditional biochemical models. We propose a method for developing mathematical models for complex systems, based on a genetic programming algorithm. The algorithm is aimed at obtaining models with a high predictive capacity, and considers an additional parameter fitting stage and an additional Jackknife stage for estimating the generalization error. We developed a model for the iron uptake system with a higher predictive capacity than classic biochemical models. This was observed both with the apical uptake dataset used for generating the model and with an independent initial rates dataset used to test the predictive capacity of the model. The model obtained is a function of time and the initial apical iron concentration, with a linear component that captures the global tendency of the system, and a non-linear component that can be associated to the movement of DMT1 transporters. The model presented in this paper allows the detailed analysis, interpretation of experimental data, and identification of key relevant components for this complex biological process. This general method holds great potential for application to the elucidation of biological mechanisms and their key components in other complex systems.

## Introduction

Iron is a trace metal, key for the development of living organisms. Its presence is necessary for several processes, such as the electron transport chain [1], oxygen transport in the blood [2], and phagocytic activity of macrophages [3], among others. The concentration of this metal must be highly controlled given that both iron excess and deficit can cause diseases, such as hemochromatosis and anemia, the latter recognized by the World Health Organization as the most common and widespread nutrition related disease [4].

Iron gets into the organism through absorption in the duodenal epithelium via the type of cell called an enterocyte. Absorption is a highly regulated process. Nevertheless, there is no controlled excretion mechanism. The only known iron loss mechanisms are due to bleeding and the exfoliation of epithelial cells. In order to maintain iron homeostasis, control mechanisms act during its absorption process.

**Fig 1:**
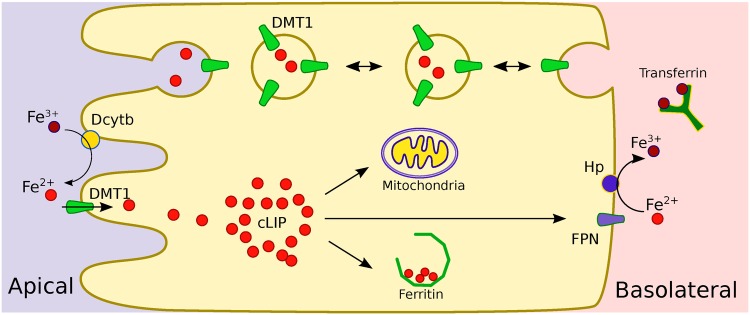
Schematic representation of the main components of the intestinal iron absorption process.

The main components of the intestinal iron absorption process are shown in Fig 1. This process can be divided into three phases: apical uptake, intracellular phase, and basolateral efflux. Non-haem iron present on the intestinal lumen can be found basically in two forms: ferrous (Fe^2+^) or ferric (Fe^3+^) ions. In the first case, iron can enter cells from intestinal lumen through the transporter protein DMT1 [5], located on the apical side (lumen) of the cells. On the other hand, prior to transport, ferric ions must first be reduced to the ferrous form by Duodenal Cytochrome b (Dcytb) [6].

After entering the cell, iron becomes part of a cytosolic pool of weakly bound iron called the cytosolic labile iron pool (cLIP) [7]. From there it can be distributed to all the cellular processes that require this metal, such as cellular respiration in the mithocondria [1], iron storage in Ferritin [8, 9], or transport outside the cell through the protein FPN1 located on the basolateral side (bloodstream) [10]. Once outside the enterocyte, iron is re-oxidized to Fe^3+^ by Hephaestin (Hp) [11] and is captured by the protein Transferrin in the interstitial fluid and plasma [12, 13].

Different iron absorption regulation mechanisms exist in order to maintain the iron concentration in the organism within appropriate ranges [14–16]. Each of them has a different level of complexity and response time. Among them, the fastest and least studied mechanism is the mucosal block, a putative process in which an initial dose of iron can reduce the absorption of a subsequent dose [16, 17]. It has been proposed that this phenomenon is due to the endocytosis of DMT1 from the enterocytes’ apical side, which has been observed to occur experimentally within 30 min after iron exposure [18]. However, only a few research papers have associated the mucosal block phenomenon with changes in iron absorption fluxes, and in all cases the experiments were performed *in vivo* [19–21].

All of the above point to the need for determining and analyzing the behavior of iron absorption fluxes in the first minutes of cellular iron exposure in highly controlled conditions, in other words, it is desirable to perform a series of experiments to characterize iron fluxes after cellular iron exposure *in vitro*. For these experiments, Caco-2 cells are considered the best model of the intestinal epithelium [22] and have been used extensively in drug absorption assays [23–25].

Due to the great relevance and complexity of the iron absorption process, a mathematical model is required in order to describe all the variables and aspects of the system that affect the amount of iron that enters an organism under different experimental conditions. In previous papers, a few models have been developed, focused on different components of this system, for instance Ferritin [26] and DMT1 [27], or the the behavior of the fluxes in the first hours after a challenge of iron [20], but none of them have modeled the fluxes of the iron absorption process in the first minutes.

A detailed knowledge of the system may help identify relevant factors for designing new therapies for iron-related diseases and assess the effects of new therapeutic drugs quickly and inexpensively [28]. Unfortunately, little information is available regarding the components and interactions involved in the mucosal block phenomenon. Hence, what is required is a method that allows creating a model based on experimental data, without knowing the system in full detail. In this paper we propose the use of the genetic programming method, which is often applied to highly complex systems, specifically when the optimum solution is expected to be highly non-linear.

Genetic programming (GP) is a branch of evolutionary algorithms (EA), which have a number of applications to optimization problems [29]. This kind of algorithm mimics Darwin’s evolutionary theory and presents two main advantages over other optimization methods: the ability to analyze many search spaces simultaneously, and the ability to solve highly complex problems with minimal information required [30–32]. The latter is due to the fact that these algorithms allow dealing with different types of objective functions in optimization problems, defining fitness functions (objective functions) that are stationary, non-stationary, continuous, discontinuous, or affected by random noise.

Due to the flexibility of this method, it is possible to make some changes to the classical algorithm in order to solve particular problems [33–35]. In the case of iron absorption, iron fluxes are determined by several factors, for instance, the intrinsic variability of the intestinal cells [36]. Hence it is necessary to consider a percentage of error in the experimental data. Therefore, the most important requirement of the model should be that it captures the general behavior of iron absorption fluxes instead of only fitting the experimental data. Following these ideas, the aim of this paper is to determine the short term effect of iron exposure on the iron absorption fluxes in Caco-2 cells and to analyze this experimental data through a mathematical model developed using genetic programming.

## Materials and Methods

### *In vitro* procedure

#### Caco-2 cell culture

Human Caco-2 cells [HTB-37, American Type Culture Collection (ATCC), Rockville, MD] were cultured in Dulbecco’s modified Eagle’s medium (DMEM) supplemented with 10% fetal bovine serum (FBS, Invitrogen-Gibco Life Technologies) at 37°C with 5% CO_2_-95% air. The cells were grown for 17 to 20 days in 12mm diameter bicameral inserts (CorningCostar). Before the start of the experiments, the transepithelial resistance (TER) was measured to assess the integrity of the monolayer. Inserts with TER below 240Ωcm^2^ were discarded [22].

#### Measurement of ^55^Fe iron fluxes

For the iron uptake determinations, the insert-grown cells were challenged with iron concentrations in apical media of 5, 10 and 20 µM ^55^FeCl_3_–ascorbate (1:20, mol:mol) in DMEM. In these experiments, Fe–ascorbate was preferred over Fe–NTA, to avoid a possible interference of Dcytb ferrireductase with the uptake process [37]. The apical iron uptake was considered as the ^55^Fe in the cells plus ^55^Fe in the basolateral medium after incubation. The experiments were performed in triplicate between 3 and 15 min of culture after the iron exposure. The cells were previously incubated overnight in a DMEM medium with 2% serum.

#### Measurement of ^55^Fe initial rates of absorption

The insert-grown cells were challenged with concentrations of 3, 5, 7, 10 and 20 µM ^55^FeCl_3_–ascorbate (1:20, mol:mol) in an apical medium. After 3 min of incubation, the apical uptake was determined. These experiments were performed under the same culture conditions as the iron uptake determination experiments.

### *In silico* procedure

In this paper, we propose a novel empirical modeling approach to capture the general behavior of iron absorption fluxes. The objective of this approach is to obtain a suitable model capable of representing the experimental data used for its fitting and, most importantly, capable of predicting new data.

Models for iron absorption fluxes were built using a symbolic non-linear regression (SNLR) process based on a genetic programming algorithm [38]. The models have two input variables: the initial iron concentration in the apical medium, and time. The output variable is the apical uptake as described above. The experimental data was divided into a training set and a test set. The training set consists of the absorption data for the first 15 min after the iron exposure, and was used for the model building process. The test set consists of the initial rates determined experimentally and was used for the final evaluation of the predictive capacity of the models. Genetic programming (GP) algorithms aim at solving complex optimization problems by establishing a parallel with the evolutionary adaptation mechanisms observed in nature. Specifically for our system, the individuals are the different models that may potentially be able to represent the experimental data. The models are mathematical expressions composed of operators (addition, multiplication, etc.), functions (cosine, sine, logarithm, etc.), variables (initial concentration and time), and numerical constants. The fitness of the individuals, *i.e.*, how well they solve the optimization problem, is given by the model’s capacity to satisfactorily represent the training dataset.

The classical GP algorithm begins with the random creation of a population of possible solutions. Then, the population of individuals (models) is evaluated through the fitness function. If the best individual in the population satisfies any of the termination conditions, then it is selected as a candidate model for that run and the algorithm ends. Otherwise, the individuals undergo a selection process. They are then recombined, mutated, or kept (elitism) to form a new population, leading to a new generation of the algorithm. This process is repeated until a termination condition is reached. A detailed description of this algorithm can be found in [38]. The parameters and inputs for the GP algorithm used in this paper were chosen following the general recommendations of [39, 40], and are shown in Table 1. A detailed explanation for each parameter can be found in [41].

**Table 1:** Parameters and criteria used in the GP algorithm.

Parameters	Value or criterion
Population size	500
Number of generations	50
Recombination probability	0.9
Mutation probability	0.1
Elitism	Keep the best
Function set	cos(), sin(), +, −, *, /, *a*^*b*^, ln(), exp()
Terminal set	Variables: *C*_0_, *t*; Constants: 1, 5, 10, 100, 1000
Initial population	Ramped-Half-and-Half
Tree depth limit	28
Selection method	Tournament
Fitness function	Jackknife Mean Square Error (*MSE*_*JK*_). See Eq (2)

For the fitness function, the implementation of a classic GP algorithm for a symbolic regression problem often uses the Mean Square Error (*MSE*) between the experimental data (the training set) and the data calculated using the model. The fitness of every model depends critically on the values of the numerical constants selected by the GP algorithm, which are not fully optimized during the training stage [42]. This has consequences, such as: low performance of the models on the training dataset and wide confidence intervals. Therefore, we added a parameter fitting stage for every model, following the concepts of the Lamarckian principle of evolution [43].

Let $\hat{Y}\left(k\right)$ be an individual in the model population generated by the GP algorithm with *k* numerical constants. Each constant is replaced by a variable parameter *β*_*k*_, generating the parameter set ***β*** = {*β*_1_, *β*_2_, …, *β*_*k*_}, and the model $\hat{Y}\left(\beta \right)$. The parameters of $\hat{Y}\left(\beta \right)$ are fitted to the experimental training dataset by minimizing the *MSE* represented in Eq (1), producing the set of fitted parameters ***β***^*MSE*^.

$$\begin{array}{c} MSE\left(\beta \right)=\sum_{i=1}^{N}\left|\hat{Y}\left(\beta \right)-Y_{i}\right| \end{array}$$ (1)

where *N* is the number of experimental observations in the training dataset.

However, models obtained by minimizing *MSE* have a tendency to overfit the experimental training dataset [44]. In such a case, the models are said to lose their generalization capacity, since they are less capable of predicting new experimental data. The generalization capacity of a model can be estimated through its generalization error (GE), *i.e.*, how badly the model performs when predicting new experimental data [44]. Therefore, in this paper we use a fitness function based on an estimation of the generalization error, considering that the lower the GE, the better the fitness of the individual.

A widely used technique to estimate the generalization error of a model is the “Jackknife”, or, “leave-one out” cross validation (LOOCV) method [35, 45]. Since the size of the dataset is modest, it is not convenient to use other re-sampling methods, like *k*-fold cross validation or the bootstrap [46]. The Jackknife method consists of repeating the parameter fitting process a number of times equal to the number of experimental observations *N*, leaving one of the experimental training data points out of each iteration, and predicting the value of the element left out using the newly found parameters. This way, on each iteration, the model’s prediction error is calculated, as well as the variation between the ***β***^*MSE*^ parameters and the ones obtained for each data subset (the partial estimate or jackknife replication). This allows obtaining an estimate of the generalization error, given by the Jackknife Mean Squared Error (*MSE*_*JK*_), at the end of the process. *MSE*_*JK*_ is defined as the sum of the differences between the experimental value *Y*_*i*_ withdrawn in iteration *i* and the value predicted by the model ${\hat{Y}}_{i}^{\left(-i\right)}$ where the experimental value *Y*_*i*_ was left out, as shown in Eq (2).

$$\begin{array}{c} MSE_{JK}=\sum_{i=1}^{N}\left|{\hat{Y}}_{i}^{\left(-i\right)}-Y_{i}\right| \end{array}$$ (2)

In addition to the GE, Jackknife allows for an unbiased estimate to be obtained for the parameter ($\beta_{k}^{*}$) of the models as well as their standard error, using Eqs (3) and (4), where *β*_*i*,*k*_ is the *k*^th^ parameter obtained in the Jackknife process when the experimental data *Y*_*i*_ was removed [47].

$$\begin{array}{c} \beta_{k}^{*}=\frac{1}{N}\sum_{i=1}^{N}\left(N\beta_{k}^{MSE}-\left(N-1\right)\beta_{i,k}\right) \end{array}$$ (3)

$$\begin{array}{c} {\hat{\sigma }}_{\beta_{k}^{*}}=\sqrt{\frac{1}{N(N-1)}\sum_{i=1}^{N}{\left(\left(N\beta_{k}^{MSE}-\left(N-1\right)\beta_{i,k}\right)-\beta_{k}^{*}\right)}^{2}} \end{array}$$ (4)

Confidence intervals for each parameter $\beta_{k}^{*}$ were calculated using Eq (5), where *t*_*α*,*ν*_ is the value of Student’s *t* for *α* = 0.05 and *ν* = *N* − *k* degrees of freedom, and $\sigma_{\beta_{k}^{*}}$ is the standard error associated to the pseudo-value.

$$\begin{array}{c} CI\left(\beta_{k}^{*}\right)=\beta_{k}^{*}\pm t_{\alpha ,\nu }{\hat{\sigma }}_{\beta_{k}^{*}} \end{array}$$ (5)

The GP algorithm was run 50 times, and every time the best model was saved. The collection of 50 best models was manually curated in order to choose the most suitable model for representing the experimental dataset. Models with a coefficient of determination lower than 0.8 and models that contradict known biochemical behavior (*e.g.*, negative concentrations, flux directions, etc.) were discarded. From the remaining set, the model with the minimum generalization error was selected as the “Best GP Model.” The coefficient of determination (*R*^2^) was calculated as follows:

$$\begin{array}{c} R^{2}=1-\frac{\sum_{i=1}^{N}{\left(Y_{i}-{\hat{Y}}_{i}\right)}^{2}}{\sum_{i=1}^{N}{\left(Y_{i}-\bar{Y}\right)}^{2}} \end{array}$$ (6)

where $\bar{Y}$ is the average of the experimental data [48].

It has been shown that *R*^2^ is an inadequate measure for the goodness of fit in non-linear models, since differences in model quality rarely affect its value more than in the third or fourth decimal place [49]. Therefore, the models will be assessed using the bias-corrected Akaike Information Criterion (AICc), a measure widely accepted for determining the validity within a cohort of non-linear models, and frequently used for model selection [50]. In contrast to *R*^2^, the lower the *AICc*, the better the data representation capacity of the model.

A comparative analysis with classical kinetic biochemical models was performed to evaluate the Best GP Model. Michaelis–Menten kinetics, Eq (7), is often used to characterize the rates of saturable mediated transport processes, due to its simplicity and effectiveness. However, some of the assumptions made in the such models are not suitable for the system under study, for example, the assumption of the presence of a single substrate and a single union site for the transporter, and of the conservation of the total amount of transporter in the system in time. Regarding the substrate, it is well known that iron is the main substrate for DMT1. However, being a symporter protein, it can be affected by the concentration of the co-substrate (H^+^) in the medium. For our studies, all experiments were performed at the same pH, so this effect can be neglected. Regardless, it is possible that the interaction of DMT1 with H^+^ increases its affinity for the main substrate, and as a result the substrate protein relationship might not remain at a 1:1 ratio as initially assumed. To take this effect into account, a Hill model was also considered, as described by Eq (8).

The Michaelis–Menten and Hill equations, Eqs (7) and (8), were used, describing the iron absorption rate as a function of time and concentration in the apical medium.

$$\begin{array}{c} \frac{d[{\text{Fe}}_{\text{in}}]}{dt}=\frac{V_{max}\left[{\text{Fe}}_{\text{out}}\right]}{K_{m}+\left[{\text{Fe}}_{\text{out}}\right]}\frac{V_{ap}}{V_{c+b}} \end{array}$$ (7)

$$\begin{array}{c} \frac{d[{\text{Fe}}_{\text{in}}]}{dt}=\frac{V_{max}{\left[{\text{Fe}}_{\text{out}}\right]}^{n}}{{\left(K_{m}\right)}^{n}+{\left[{\text{Fe}}_{\text{out}}\right]}^{n}}\frac{V_{ap}}{V_{c+b}} \end{array}$$ (8)

The Michaelis–Menten model in Eq (7) has two parameters: the maximum iron transport velocity *V*_*max*_ and the Michaelis constant *K*_*m*_. *V*_*max*_ is strongly dependent on several factors such as the number of transporter proteins, temperature and pH, while *K*_*m*_ depends mainly on the intrinsic characteristics of the proteins under study [51]. Previous results have reported *K*_*m*_ ≈ 7 µM for the iron transport system [52]. The Hill model in Eq (8) has three parameters: *V*_*max*_, *K*_*m*_ and the Hill constant *n*, which is a measure of the molecular cooperativity in the transport process. A value of the Hill constant *n* > 1 indicates a cooperative process for molecular transport, while *n* < 1 indicates a competitive one [53].

The iron absorption rate in Eqs (7) and (8) is affected by the ratio between the volume of the apical medium (*V*_*a*_) and the cellular and basolateral media (*V*_*c*+*b*_), as these parameters affect the iron concentration in the transport process. Based on our experimental conditions and the reported characteristics of Caco-2 cells, we assumed *V*_*a*_ = 200 µL and *V*_*c*+*b*_ = 1000 µL.

All calculations were performed using MATLAB^®^ [54]. The freely available GPLAB MATLAB implementation of the GP algorithm was used to perform all simulations [55]. Experimental data (training and test data sets) are available in the S1 and S2 Tables in the supplementary information section. The MATLAB^®^ code is available upon request.

## Results and Discussion

### Kinetics of iron uptake in Caco-2 cells

**Fig 2:**
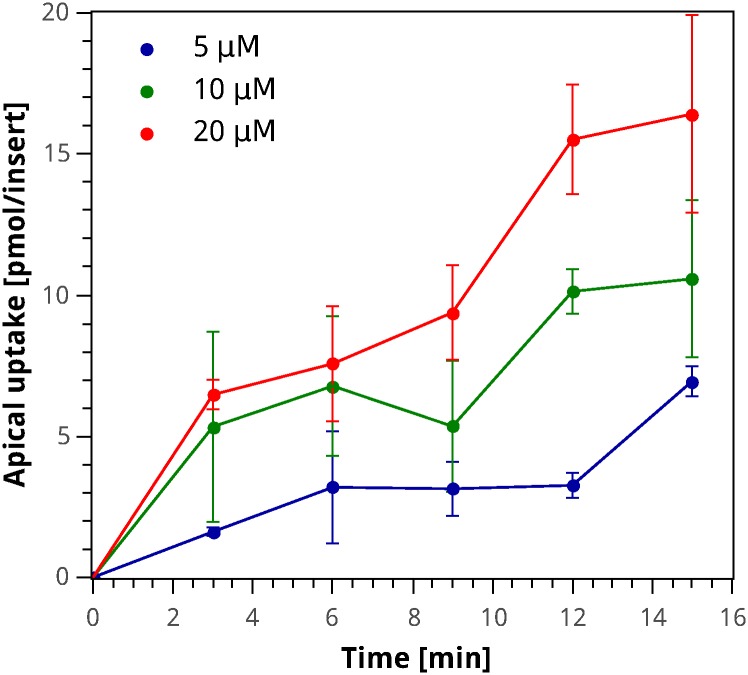
Apical uptake experimental data for different iron challenge concentrations. Amount of iron transported into the cell over time after an iron challenge of 5 µM (blue), 10 µM (green) or 20 µM (red) in the apical medium. Circles correspond to the average value of the sample and error bars indicate its standard deviation.

**Fig 3:**
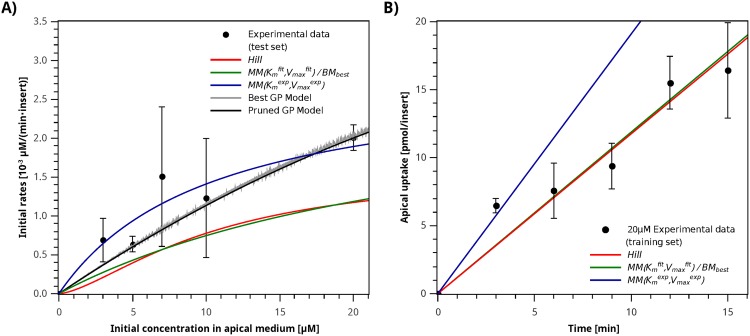
Initial rates and apical uptake experimental data and model simulations. **A)** Initial iron absorption rates. **B)** Apical iron uptake after a 20 µM iron challenge. Circles correspond to the average value, error bars indicate standard deviation for each sample. $MM\left(K_{m}^{fit},V_{max}^{fit}\right)/BM_{best}$ best biochemical model simulation (green line); Hill model (red line); $MM(K_{m}^{exp},V_{max}^{exp})$ model (blue line); Best GP Model (gray line) and Pruned GP Model (black line).

The amount of iron entering the cell was measured for initial iron concentrations of 5, 10 and 20 µM in the apical media, for 15 min after the iron exposure. Fig 2 shows that in an apical medium with a larger iron concentration, there is a larger iron uptake by the cells. Between 3 and 12 min after the iron exposure, there is a significant decrease in the rate of iron absorption compared to its initial value. Nevertheless, during the next five minutes, the rate increases again. This behavior is observed for the three initial apical iron concentrations. The experimental patterns observed in the absorption rates over time for the three extracellular iron concentrations studied drift away from the standard behavior of a transport system that could be described using a Michaelis–Menten or Hill type of expression. This behavior can be attributed to the variation in the amount of DMT1 present in the apical membrane after the iron exposure, as suggested by Nuñez *et al.* [18]. As reported in the literature, Caco-2 cells have a large natural variability between cultures [36], which results in the substantial standard deviation observed in Figs 2 and 3A.

The initial iron absorption rates were determined for the first three minutes, for apical iron concentrations of 3, 5, 7 and 20 µM. A higher iron absorption rate with a higher initial apical iron concentration can be observed, as shown in Fig 3A. A Michaelis–Menten model, Eq (7), was fitted to this experimental data. The Michaelis constant $K_{m}^{exp}=10.36µ\text{M}$ and maximum velocity $V_{max}^{exp}=0.0145µM\;{\text{min}}^{-1}$ were obtained. Experimental values for *K*_*m*_ for DMT1 have been previously reported at different pHs [52]. The value of *K*_*m*_ reported by Linder *et al.* at pH of 7 is $K_{m}^{Linder}=7µM$ [52], slightly lower than the one calculated directly from our experimental data. This difference can be attributed to the high incubation time (20 min) used to obtain the reported values in [52], which would underestimate the value for *K*_*m*_ compared to standard initial velocity experiments like the ones performed in this research, which use incubation times on the order of 3–15 min.

The simulation results for the initial rates and apical uptake are shown in Fig 3A and 3B, respectively. It can be observed that the Michaelis–Menten model with parameters $K_{m}^{exp}$ and $V_{max}^{exp}$ can describe reasonably well the experimental data for the initial rate, but perform poorly on the apical uptake experimental dataset. In fact the coefficients of determination obtained on these datasets are $R_{Test}^{2}=0.645$ and $R_{Train}^{2}=-0.550$ for the initial rate dataset and the apical uptake dataset, respectively. The negative coefficient of determination indicates that the average experimental data value represents the experimental data better than the model. Since $K_{m}^{exp}$ is of the same order of magnitude as the values reported in the literature, the model’s overestimation of the apical iron uptake could be due to an overestimation of the value for the parameter $V_{max}^{exp}$. The mucosal block phenomenon described in [16, 17] would decrease the amount of DMT1 in the apical membrane, therefore the overestimation of $V_{max}^{exp}$ could be due to the reduction in the amount of DMT1 available for iron transport.

### Classic biochemical transport models

In order to establish a baseline to study the models generated by the proposed GP algorithm, the generalization capacities of the Michaelis–Menten and Hill models were determined for the training experimental dataset. Four models were generated to fit the training dataset, considering combinations of parameters reported in the literature ($K_{m}^{Linder}$ [52]), experimentally determined parameters ($K_{m}^{exp}$), and parameters obtained from fitting the Michaelis–Menten and Hill models to the training dataset ($V_{max}^{fit}$, $K_{m}^{fit}$, *n*^*fit*^) as described in Table 2. The parameter fitting results for each model on the training set as well as the coefficient of determination for both datasets, the *AICc* indicator, and the generalization error of each model, are also shown in Table 2. The simulation results for iron uptake at 20 µM apical iron concentration for the three Michaelis–Menten based models are shown in Fig 3B.

**Table 2:** Statistical assessment of classic biochemical models.

					$R_{Train}^{2}$		$R_{Test}^{2}$		*AICc*_*Train*_		*AICc*_*Test*_		
Model	Parameters*	$\beta_{k}^{MSE}$	$\beta_{k}^{*}$	CI**($\beta_{k}^{*}$) (95%)	$\beta_{k}^{MSE}$	$\beta_{k}^{*}$	$\beta_{k}^{MSE}$	$\beta_{k}^{*}$	$\beta_{k}^{MSE}$	$\beta_{k}^{*}$	$\beta_{k}^{MSE}$	$\beta_{k}^{*}$	GE***
Hill	$K_{m}^{fit}$	1.05 × 10^1^	5.45	± 6.58 × 10^1^	0.816	0.760	0.135	0.210	1.88 × 10^2^	2.03 × 10^2^	5.02 × 10^1^	4.87 × 10^1^	1.823
	$V_{max}^{fit}$	8.09 × 10^−3^	6.02 × 10^−3^	± 3.35 × 10^−2^									
	*n*^*fit*^	1.52	1.46	± 7.04									
$MM(K_{m}^{fit},V_{max}^{fit})$	$K_{m}^{fit}$	2.85 × 10^1^	2.41 × 10^1^	± 1.46 × 10^2^	0.813	0.724	0.150	0.151	1.86 × 10^2^	1.87 × 10^2^	4.65 × 10^1^	4.65 × 10^1^	1.820
	$V_{max}^{fit}$	1.44 × 10^−2^	1.28 × 10^−2^	± 5.19 × 10^−2^									
$MM(K_{m}^{Linder},V_{max}^{fit})$	$K_{m}^{Linder}$ [52]	7.00	-	-	0.754	0.754	0.199	0.200	2.02 × 10^2^	2.02 × 10^2^	4.55 × 10^1^	4.54 × 10^1^	2.106
	$V_{max}^{fit}$	6.99 × 10^−3^	7.00 × 10^−3^	± 4.11 × 10^−3^									
$MM(K_{m}^{exp},V_{max}^{fit})$	$K_{m}^{exp}$	1.04 × 10^1^	-	-	0.784	0.784	0.194	0.195	1.95 × 10^2^	1.95 × 10^2^	4.56 × 10^1^	4.55 × 10^1^	1.923
	$V_{max}^{fit}$	8.23 × 10^−3^	8.23 × 10^−3^	± 4.46 × 10^−3^									
$MM(K_{m}^{exp},V_{max}^{exp})$	$K_{m}^{exp}$	1.04 × 10^1^	-	-	-0.550	-	0.645		3.01 × 10^2^		3.08 × 10^1^		-
	$V_{max}^{exp}$	1.45 × 10^−2^	-	-									

* Parameters units:

*K*_*m*_: [µM]

*V*_*max*_: [µM min^−1^]

** Confidence Interval

*** Generalization Error

The assessment of the representation capacity of the models obtained using the coefficient of determination *R*^2^ and the *AICc* indicator are consistent for all the models studied. For instance, in Table 2 columns $R_{Train}^{2}$ ($\beta_{k}^{MSE}$) and *AICc*_*Train*_ ($\beta_{k}^{MSE}$) show that the higher the value of *R*^2^ the lower the value of the corresponding *AICc*. Hence, from now on, all the discussions referring to the models’ performance will be based on the coefficient of determination only.

As can be seen in Table 2, for all models, the confidence intervals obtained for every parameter are large, in some cases even exceeding the value of their respective parameters. Furthermore, the parameters obtained by fitting the experimental training dataset ($\beta_{k}^{MSE}$) and the ones obtained using the Jackknife method ($\beta_{k}^{*}$) are quite different from each other, which may lead to a significant difference in the performance of these models, both on the training dataset and the test dataset. More specifically, the models that use the unbiased parameters provided by Jackknife validation ($\beta_{k}^{*}$) reach higher *R*^2^ in the test dataset, indicating a better predictive capacity.

The $MM(K_{m}^{fit},V_{max}^{fit})$ model has a higher *R*^2^ on the iron absorption fluxes dataset than the $MM(K_{m}^{exp},V_{max}^{exp})$ model; however the *K*_*m*_ value obtained in this case is considerably higher than in the $MM(K_{m}^{exp},V_{max}^{exp})$ model ($K_{m}^{fit}=28.45$ vs. $K_{m}^{exp}=10.36$), resulting in significantly lower values for the predicted initial rates, which explains the low *R*^2^ values obtained for the initial rate dataset. It is important to take into account that the generalization error for $MM(K_{m}^{fit},V_{max}^{fit})$ is 1.82, which sets a lower bound for the expected generalization capacity of the models generated with the GP algorithm.

On the other hand, $MM(K_{m}^{Linder},V_{max}^{fit})$ and $MM(K_{m}^{exp},V_{max}^{fit})$ have similar GEs and coefficients of determination for both datasets. In both cases, the value of the maximum velocity obtained, $V_{max}^{fit}$, is approximately 50% that of the $V_{max}^{exp}$ determined experimentally from the data for the initial rates. Given that the *K*_*m*_’s for both models are similar, *V*_*max*_ is the only parameter that controls the slope of the initial rates curve—see Eq (7). Since $V_{max}^{fit}$ is lower than $V_{max}^{exp}$, this leads to an underestimation of the initial rates. In addition, both models exhibit a worse generalization capacity than the $MM(K_{m}^{fit},V_{max}^{fit})$ model.

The value of $V_{max}^{fit}$ obtained for the Hill model also leads to an underestimation of the initial rates. However, the *K*_*m*_ for the Hill model is similar to the one obtained experimentally ($K_{m}^{exp}=10.36µM$), allowing a better performance of this model in terms of *R*^2^, both for the training and test sets. The generalization error and coefficient of determination in the training set obtained by the Hill model are similar to the best Michaelis–Menten model ($MM(K_{m}^{fit},V_{max}^{fit})$), but the Michaelis–Menten model has slightly better performance on the test dataset; it also has one less parameter than the Hill model and narrower confidence intervals (CI). Taking all this into consideration, $MM(K_{m}^{exp},V_{max}^{fit})$ was selected as the best model obtained from the Michaelis–Menten and Hill equations, and will be referred to as the *Best Biochemical Model* (*BM*_*best*_).

Simulation results for the best classic biochemical model, both for the training (Fe 20 µM) and test datasets, are shown in Fig 3B and 3A, using the parameters $\beta_{k}^{MSE}$. This model provides a suitable representation of the training data. But it performs poorly on the test dataset, as it underestimates the initial rate for most concentrations. This poor performance is confirmed by the indicators presented in Table 2, where the coefficient of determination between the model and the experimental data reaches values below 0.4 for the test dataset.

The model linearly follows the general increasing trend observed for the experimental data. However, the distribution of the experimental results over time suggests changes in the iron absorption velocity during the experiments. This characteristic can not be captured by either the Michaelis–Menten model or the Hill model. Therefore, a model capable of representing greater complexity is required.

### Genetic Programming Models

#### Procedure for the selection of the best model obtained by genetic programming

The genetic programming algorithm was run 50 times, starting from different initial populations. The best model was selected for each run, based on its generalization error (the lower the better), generating a set of 50 GP candidate models. Models that contradict known biochemical behavior, such as iron concentrations and iron absorption velocities being greater than or equal to zero, were discarded. In addition, candidate models whose functions exhibited singularities at some point in the domain of the variables (*t* ≥ 0 min and *C*_0_ ≤ 20 µM) were also removed from the set of candidate GP models. In this way, a final set of ten candidate models was obtained. The candidate model with the best coefficient of determination in the training dataset was selected as the best model generated by the GP algorithm (“Best GP Model”).

**Fig 4:**
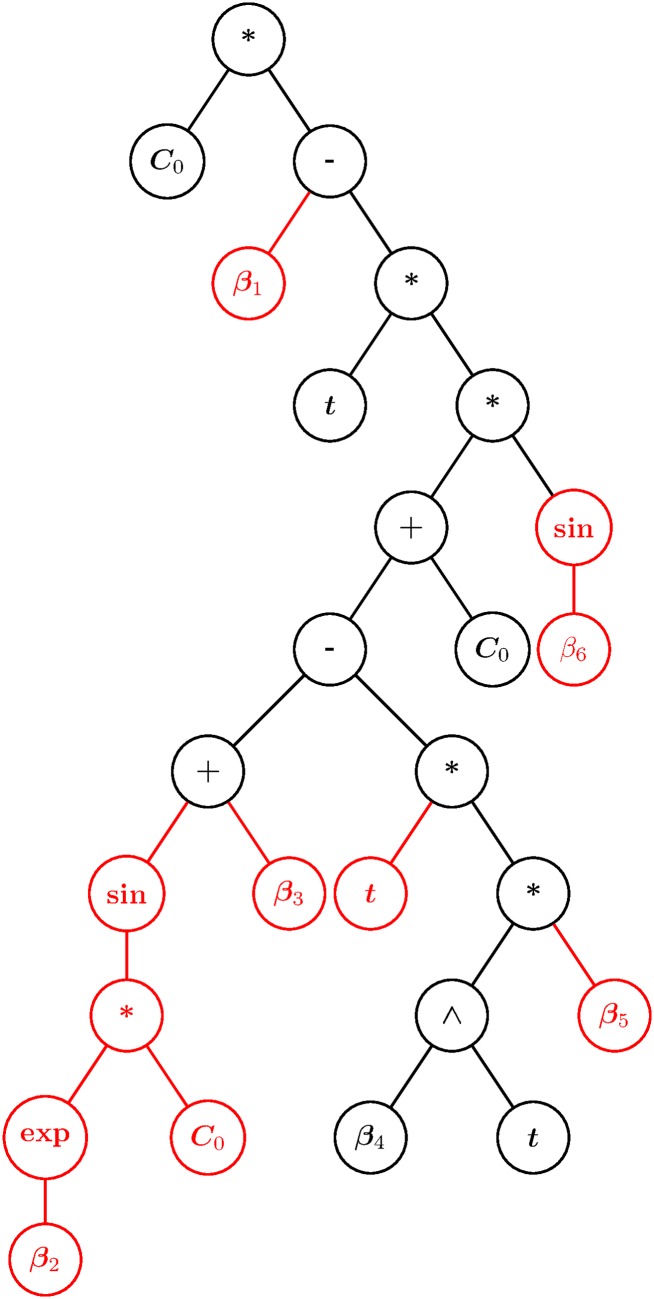
Tree representation of the Best GP Model. Black branches and leaves are conserved in the subpopulation with best fitness. Red branches and leaves present different structures and parameters in the subpopulation.

The Best GP Model is represented by Eq (9) and the tree in Fig 4. In Eq (9), *Ap*_*Up*_ represents the apical iron uptake in Pmolinsert^−1^, *C*_0_ corresponds to the initial apical iron concentration in µM, *t* is the time in minutes, and *β*_*i*_ are the fitted parameters. The tree in Fig 4 is an equivalent computational representation of Eq (9), where the nodes represent the mathematical operations, variables, and parameters found in the equation.

Table 3 shows that the coefficient of determination between the model and the experimental iron absorption data and between the model and the initial rates data are *R*^2^ = 0.849 and *R*^2^ = 0.561, respectively. The validation results for the model using the Jackknife method are characterized by narrow confidence intervals for the model’s parameters and a low generalization error, as shown in Table 3. All parameters are identified as significant at 95% confidence levels, since all the *p*-values are lower than 0.05 (*t*-test).

$$\begin{array}{c} Ap_{Up}\left(C_{0},t\right)=\beta_{1}\cdot C_{0}-sin\left(\beta_{6}\right)\cdot t\cdot C_{0}\cdot (C_{0}+\beta_{3}+sin\left(C_{0}\cdot exp\left(\beta_{2}\right)\right)-\beta_{5}\cdot t\cdot {\beta_{4}}^{t}) \end{array}$$ (9)

**Table 3:** Main Jackknife validation results for the Best GP Model.

**Parameter**	$\beta_{k}^{MSE}$	$\beta_{k}^{*}$	**Confidence Intervals** (*α* = 0.05)	***p*-value**
*β*_1_	-7.93 × 10^−3^	-7.10 × 10^−3^	± 1.38 × 10^−3^	6.11 × 10^−85^
*β*_2_	2.15 × 10^2^	2.15 × 10^2^	± 4.06 × 10^−5^	0
*β*_3_	-6.25 × 10^1^	-8.57 × 10^1^	± 1.15 × 10^1^	5.76 × 10^−85^
*β*_4_	4.39 × 10^−1^	4.34 × 10^−1^	± 5.03 × 10^−3^	2.17 × 10^−138^
*β*_5_	1.39 × 10^2^	1.76 × 10^2^	± 2.24 × 10^1^	1.64 × 10^−86^
*β*_6_	1.01 × 10^2^	1.01 × 10^2^	± 3.85 × 10^−4^	8.09 × 10^−295^
$R_{Train}^{2}\left(\beta_{k}^{MSE}\right)$	$R_{Train}^{2}\left(\beta_{k}^{*}\right)$	$R_{Test}^{2}\left(\beta_{k}^{MSE}\right)$	$R_{Test}^{2}\left(\beta_{k}^{*}\right)$	***M*** *SE*_*jk*_
0.849	0.382	0.561	0.432	1.46
$AICc_{Train}\left(\beta_{k}^{MSE}\right)$	$AICc_{Train}\left(\beta_{k}^{*}\right)$	$AICc_{Test}\left(\beta_{k}^{MSE}\right)$	$AICc_{Test}\left(\beta_{k}^{*}\right)$	
1.86 × 10^2^	2.61 × 10^2^	5.21 × 10^1^	5.67 × 10^1^	

#### Analysis of population diversity and convergence of the GP algorithm

**Fig 5:**
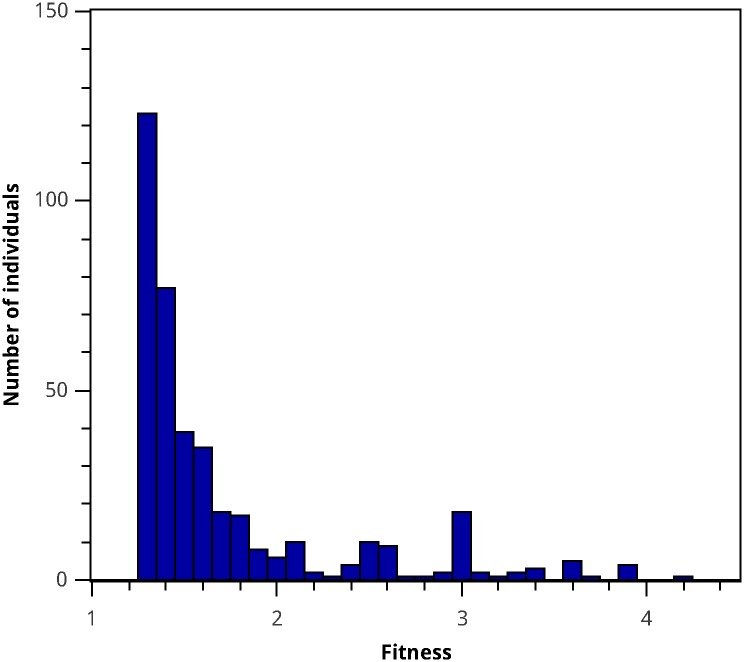
Final fitness distribution of the population where the Best GP Model appeared. The fitness distribution was calculated for the 400 best individuals of the final generation of the run where the Best GP Model was obtained.

In order to determine whether there are relevant patterns in the individual’s population, the best model and the last generation of that specific run were analyzed. Fig 5 presents a histogram for the number of individuals versus their fitness, showing that a large percentage of the population has a fitness close to or equal to the best model’s fitness. Moreover, when calculating the population’s *variety* to study the final population’s diversity [38], we observed that only 48% of the population genotypes were unique. This can be explained by the fact that there are a large number of copies of the best individual in the population (24.6% of the total population). But this could also be due to individuals with a fitness close to the Best GP Model sharing key features with it. In order to identify the main characteristics of the models closely related to the Best GP Model, the genotype of the best individual was compared to the subpopulation’s genotype within a 10% fitness range. This allowed the identification of conserved structural motifs in models that exhibit good fitness. The results are shown in Fig 4, where over the tree of the Best GP Model, the structural motifs conserved in all individuals of the studied subpopulation are shown in black, while structural changes both in branch structure as well as in leaf values are highlighted in red. Of the nodes of the Best GP Model tree, 57.7% are conserved. The conserved nodes include all of the model’s variables, parameters, basic mathematical operations, and an exponential type term $\beta_{4}^{t}$, which is the only non-linear term conserved. The non-conserved branches are an exclusive characteristic of the Best GP model. They include all the other parameters and a sinusoidal term dependent on the initial apical iron concentration *C*_0_, which is also a non-linear term. In what follows, we analyze the effect of each of these terms on the model’s characteristics.

**Fig 6:**
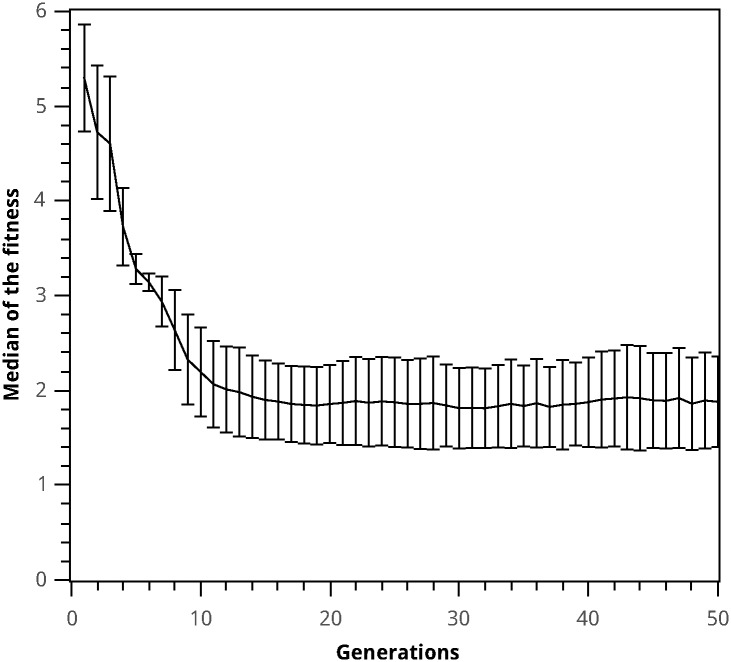
Evolution of the median of the fitness as a function of the generation number. The median of the fitness and its standard deviation was calculated for each run of the genetic programming algorithm throughout 50 generations.

In order to analyze the convergence towards a solution, the median of the fitness for each generation in every run was determined. The results are shown in Fig 6. It can be observed that the population converges after the 20th generation, reaching a median fitness that is 32% of the initial population’s median fitness. The largest reduction in the median fitness is observed in the first ten generations. This indicates that a choice of generation number equal to 50 generations is sufficient to achieve convergence.

#### Structural analysis of the Best GP Model obtained

Both in Eq (9) and in Fig 4, the presence of highly nonlinear components such as sine functions and exponentials can be identified, in agreement with the non-linear characteristics observed in our experimental data. Each of the non-linear components in Eq (9) was selected by the GP algorithm through its evolutionary mechanism, in order to provide the model with the maximun generalization capacity. Each of these terms plays a different role in the main model, in varying degrees, therefore, in order to gain a deeper insight into the model, a relation between each term in Eq (9) and its effect on the obtained fluxes and velocities must be established.

The model in Eq (9) has terms that depend on the initial apical iron concentration *C*_0_ and time *t*. To facilitate the analysis, *C*_0_ was assumed to be constant and only terms that depend on time were considered to be variable. With this, Eq (9) becomes Eq (10), where *a*_*i*_ are lumped parameters defined to facilitate the analysis.

$$\begin{array}{c} {Ap_{Up}\left(t\right)|}_{C_{0}=Const}=a_{0}+a_{1}t+a_{2}\cdot t^{2}\cdot a_{3}^{t}\;a_{i}>0\;\forall i \end{array}$$ (10)

**Fig 7:**
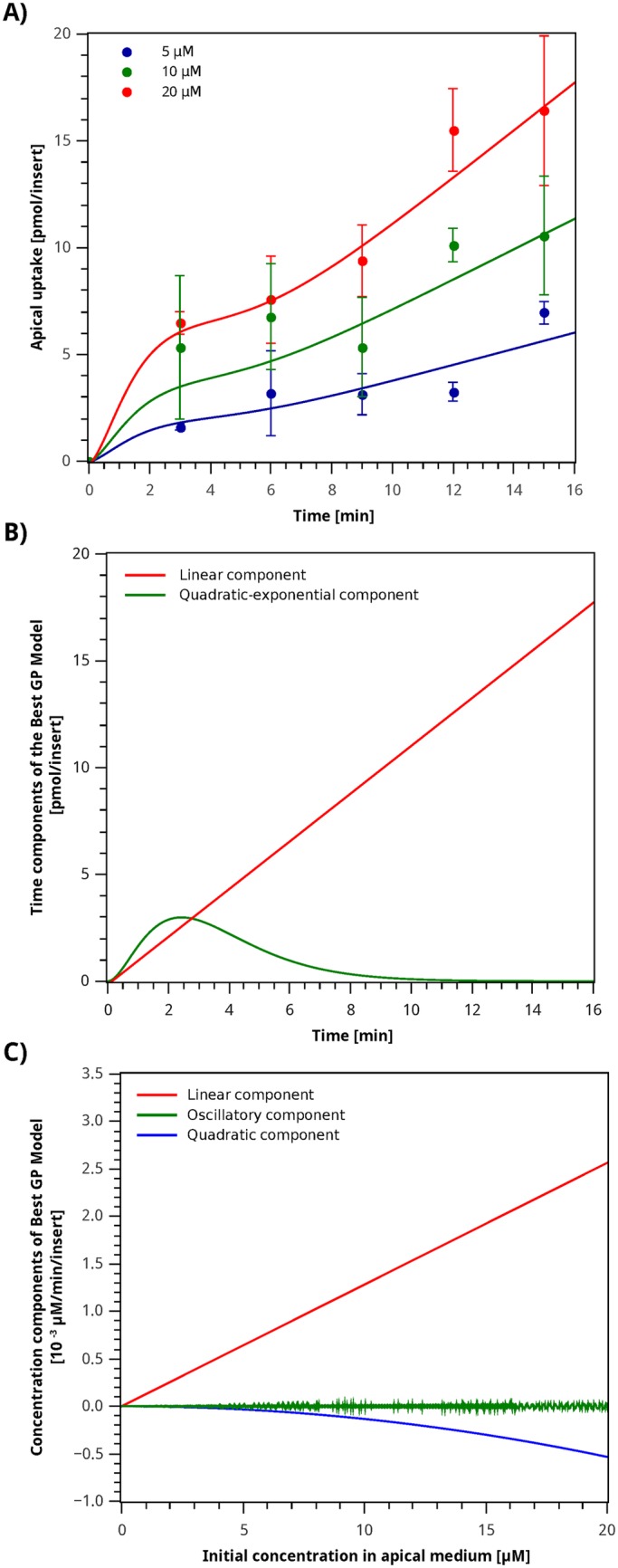
Best GP model simulation of the apical iron uptake and study of its mathematical components. **A)** Simulation and experimental data. Circles correspond to the average value, error bars indicate standard deviation for each sample, and the curve plotted corresponds to simulation results for the model described by *Ap*_*Up*_, Eq (9). **B)** Time components of Best GP Model. The curves plotted correspond to simulation results for the components of the model described by Eq (10) with *C*_0_ = 20 µM. (red) linear component and (green) quadratic-exponential component. **C)** Concentration components of Best GP Model. The curves plotted correspond to simulation results for the components of the model described by Eq (11). (red) linear component; (green) oscillatory component and (blue) quadratic component.

Two components can be identified in Eq (10): a linear term *a*_0_ + *a*_1_*t* and a quadratic contribution combined with an exponential expression $a_{2}t^{2}a_{3}^{t}$. Fig 7B shows the simulation results for each of these terms over time. Even though the linear component is responsible for the system’s overall dynamics, the quadratic-exponential component captures the more interesting complexity of the system, exhibiting a bell-shaped behavior. The quadratic-exponential component is responsible for the variation in iron absorption velocity during the first minutes after the iron challenge. In consequence, it is responsible for the difference observed in the absorption profiles obtained by the Best GP Model and the quasi-linear profiles obtained by the Hill and Michaelis–Menten models. This term accounts for the variation in the content of DMT1 on the apical membrane previously reported in the literature [18], as a result of the movement of DMT1 from the membrane to the cytoplasm after an iron challenge.

The effect of concentration in Eq (9) was studied by keeping the time constant and equal to *t* = 3 min, which is the final time point of the initial rate studies. With this, Eq (9) can be reformulated as Eq (11), which has three main components: a linear component *a*_1_*C*_0_, a quadratic component $-a_{2}C_{0}^{2}$, and a sinusoidal component *a*_3_ ⋅ *C*_0_ ⋅ sin(*a*_4_ ⋅ *C*_0_).

$$\begin{array}{c} {Ap_{Up}\left(C_{0}\right)|}_{t=Const}=a_{1}\cdot C_{0}-a_{2}\cdot C_{0}^{2}+a_{3}\cdot C_{0}\cdot \sin\left(a_{4}\cdot C_{0}\right)\;a_{i}>0\;\forall i \end{array}$$ (11)

The simulation results for each component in Eq (11) are shown in Fig 7C. It can be observed that the contribution of the quadratic and linear terms in Eq (11) is much larger than that of the sinusoidal term. The sinusoidal component only introduces small oscillations as a function of the iron concentration. For instance, at Fe 20 µM, this term represents only 0.96% of the total iron absorption velocity. In addition, this term is not a conserved branch on the sub-population of models with better fitness. Hence, the sinusoidal term does not contribute meaningfully to either the data representation ability of the model or to its generalization capacity, and therefore can be removed from the model.

Based on the previous discussion, we propose a Pruned GP Model, defined as the Best GP Model after the branch containing the sine function is removed: Eq (12).

$$\begin{array}{c} Ap_{Up}\left(C_{0},t\right)=\beta_{1}\cdot C_{0}-sin\left(\beta_{6}\right)\cdot t\cdot C_{0}\cdot \left(C_{0}+\beta_{3}-\beta_{5}\cdot t\cdot \beta_{4}^{t}\right) \end{array}$$ (12)

The simulation results for the iron absorption and initial rates for this model are shown in Figs 7A and 3A, respectively. The coefficients of determination and GE values for the Pruned GP Model are shown in Table 4. It can be observed that the coefficients of determination for the pruned model are better than those obtained for the Best GP Model (Table 3). Nevertheless, its generalization error (GE) is slightly higher (7.5%), which explains why the GP algorithm did not select the Pruned GP Model in the first place. This highlights the relevance of analyzing the models obtained with the GP algorithm since, even though the algorithm delivers a good model in statistical terms, it can be improved by taking into account biological considerations specific to the system under study.

**Table 4:** Main Jackknife validation results for the Pruned GP Model.

**Parameter**	$\beta_{k}^{MSE}$	$\beta_{k}^{*}$	**Confidence Intervals** (*α* = 0.05)	**p-value**
*β*_1_	8.79 × 10^−3^	9.03 × 10^−3^	± 6.75 × 10^−2^	1.73 × 10^−15^
*β*_3_	-5.40 × 10^1^	-4.88 × 10^1^	± 2.28 × 10^1^	3.52 × 10^−74^
*β*_4_	4.39 × 10^−1^	4.46 × 10^−1^	± 1.75 × 10^−1^	2.22 × 10^−75^
*β*_5_	1.48 × 10^2^	4.133 × 10^1^	± 2.48 × 10^2^	1.28 × 10^−44^
*β*_6_	1.01 × 10^2^	1.01 × 10^2^	± 9.12 × 10^−4^	1.65 × 10^−307^
$R_{Train}^{2}\left(\beta_{k}^{MSE}\right)$	$R_{Train}^{2}\left(\beta_{k}^{*}\right)$	$R_{Test}^{2}\left(\beta_{k}^{MSE}\right)$	$R_{Test}^{2}\left(\beta_{k}^{*}\right)$	***M*** *SE*_*jk*_
0.854	0.646	0.575	0.610	1.57
$AICc_{Train}\left(\beta_{k}^{MSE}\right)$	$AICc_{Train}\left(\beta_{k}^{*}\right)$	$AICc_{Test}\left(\beta_{k}^{MSE}\right)$	$AICc_{Test}\left(\beta_{k}^{*}\right)$	
1.81 × 10^2^	2.28 × 10^2^	4.60 × 10^1^	4.44 × 10^1^	

The simulation results for the initial rates (test set) obtained by the Pruned GP Model are shown in Fig 3A. The overall trend of the Pruned GP Model is similar to those of the Michaelis–Menten (green line) and Hill (red line) models. However, the initial rates obtained by the Pruned GP Model (gray line) are in good agreement with the average experimental data in the test set, which is noteworthy, as this dataset was not used to build this model. Removing the branch associated to the sinusoidal term slightly improves the coefficient of determination, from *R*^2^ = 0.561 to *R*^2^ = 0.575, and reduces the number of parameters, while eliminating the high frequency oscillations introduced by this term. In addition, the Pruned GP Model exhibited a slight increase in the coefficient of determination for describing iron absorption fluxes (training set), which considered along with the small change observed in the jackknife error indicates a negligible increase in the model’s overfitting.

#### Analysis of the domain of validity of the Pruned GP Model

**Fig 8:**
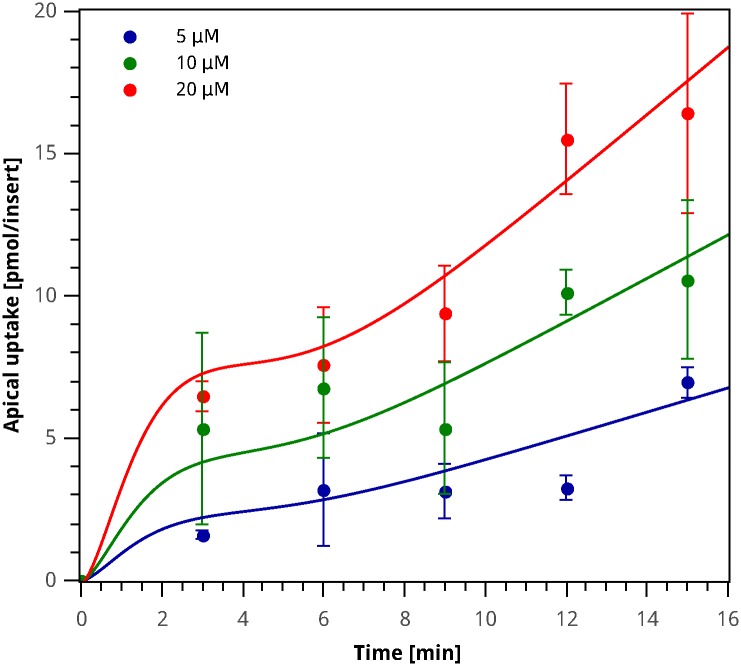
Apical iron uptake experimental data and Pruned GP Model simulation. Circles correspond to the average value, error bars indicate standard deviation for each sample, and the curve plotted corresponds to simulation results for the model described by Eq (12).

As can be observed in Fig 8, for the simulations of the training and test datasets, the Pruned Model satisfies the biological restrictions imposed, that is, positive concentrations and absorption velocities for the first 15 min of simulation. In addition, for the test dataset (see Fig 3A), the initial rates increase as the initial iron concentration in the apical media is increased, as expected for the transport system under study. Based on these observations, we consider this model to capture the biological characteristics of the system under study for the time and concentration intervals assessed experimentally.

**Fig 9:**
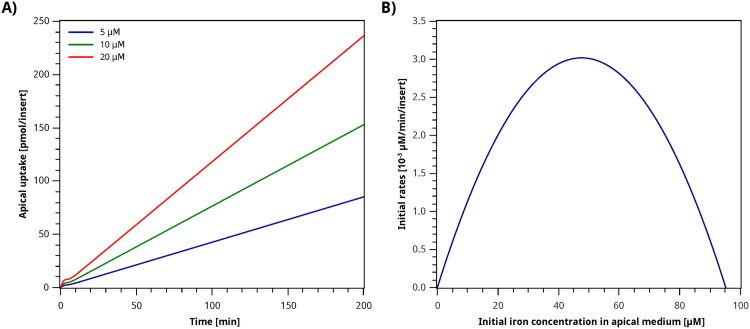
Study of the domain of validity of the Pruned GP Model. **A)** Long term simulation of apical iron uptake. **B)** Simulation of initial rates up to high iron concentrations in the apical medium.

In order to examine the performance of this model outside the experimental range, simulations were performed for longer time and concentration intervals. Fig 9A and 9B show the iron absorption fluxes up to 200 min, and the initial velocities for up to 100 µM initial apical iron concentration, respectively. It can be observed in Fig 9A that for longer times the model follows a linear trend as a function of time, without reaching a steady state. For our experimental setting, changes in the apical iron concentration are negligible, and it can therefore be considered as constant. As a result, the model is a function of the initial iron concentration *C*_0_, and does not account for changes in iron concentration as a result of the transport process. This characteristic restricts the model’s use to simulation times where the iron concentrations are negligible.

As shown in Fig 9B, the initial rates reach a maximum at 48 µM initial apical iron concentration, and then decrease to zero *ca.* 96 µM. The initial rate is expected to reach a maximum when the transport system saturates with iron, but for iron concentrations higher than the saturation point, it should remain at its maximum. The maximum value observed in Fig 9B is 3.02 × 10^−3^ µM min^−1^insert^−1^, and can be interpreted as similar to the parameter *V*_*max*_ in the Michaelis–Menten equation. In this case, the value for the maximum initial rate is lower than what was obtained for *V*_*max*_ in the Michaelis–Menten models (see Table 2). This feature of the Pruned GP Model is coherent with the mucosal block phenomenon, where the system’s *V*_*max*_ would decrease as a result of the reduction of the amount of DMT1 transporters in the membrane.

Based on the discussion presented above, the domain for the variables of the Pruned GP Model, *t* and *C*_0_, where the model behaves adequately in biological terms, corresponds to *C*_0_ ∈ [0, 50] µM and *t* within the range where the apical iron concentration can be considered as constant.

#### Parameters and statistical analysis of the Pruned GP Model

Note that the Pruned GP Model shown in Eq (12) has five parameters, while the Michaelis–Menten and Hill models have two and three parameters, respectively. Since the Pruned GP Model has a larger number of parameters, it has fewer degrees of freedom, and can therefore be expected to perform better on the training dataset (*e.g.*, a higher *R*^2^). However, the Pruned GP Model not only has a larger determination coefficient but also exhibits a lower generalization error, which suggest a greater predictive capacity than the Michaelis–Menten and Hill models. In fact, this can be observed in the simulation of the test dataset as shown in Fig 3A, where, unlike the other models, the Pruned GP Model curve goes through the experimental data points.

To analyze the statistical significance of each of the parameters in the Pruned GP Model, the *p*-value for each of them was calculated (see Table 4). All parameters are identified as significant at 95% confidence levels, since all *p*-values are lower than 0.05 (*t*-test).

As shown in Table 4, the fitted parameters $\beta_{k}^{*}$, which are calculated as the average parameter on each iteration during the validation stage, are within the same order of magnitude as the $\beta_{k}^{MSE}$. This indicates that the parameter set can be estimated precisely for the experimental dataset [56], making the model robust and more resistant to experimental errors.

### Final remarks

The models generated by the genetic programming algorithm perform better, in statistical terms, on the training dataset and exhibit a better predictive capacity on the test dataset. It must be noted that the analysis of the mathematical expression of the Pruned GP Model suggests specific biological features for the experimental system in the experimental time and concentration domains, that can be associated to the movement of DMT1 from the membrane. However, the Hill and Michaelis–Menten models show a more biologically sound behavior at high iron concentrations and longer simulation times. This is due to the fact that, unlike the models generated by genetic programming, they have a mechanistic base that assumes a constant amount of transporter on the membrane.

On the other hand, the models generated by genetic programming allow representing the experimental data without a detailed knowledge of the phenomenon. In addition, their study allows obtaining a deeper insight into the relevant components in describing the phenomenon, for instance, changes in iron absorption velocities observed in time that might be associated to changes in the amount of transporters present in the membrane as a result of mucosal block. This study poses new questions regarding the system under study in terms of the transport mechanisms, transporter internalization, key factors controlling this process, and its dependence on extracellular iron levels.

## Conclusions

Iron absorption fluxes in Caco-2 cells were determined experimentally, and a mathematical model was developed that allows predicting the amount of iron entering the cell at a given time, considering different initial iron concentrations in the intestinal lumen (apical side). The model was developed using a symbolic nonlinear regression process based on a genetic programming algorithm with two additional stages: a parameter optimization and measurement of a generalization error. These additional steps allowed obtaining better confidence intervals for the model’s parameters in the studied functions and reducing the generalization error, thereby increasing the predictive capacity of the model. The model obtained can accurately represent the experimental data and captures the main characteristics of the biological phenomenology of the system.

Experimental data reveal a complex dynamic in the iron absorption process, which is reflected in the noticeable changes in apical iron uptake observed. This complex dynamic could be associated to the interaction between iron and the DMT1 transporter and to previously reported phenomena, namely the internalization of DMT1 transporters and the mucosal block. Therefore, the iron internalization mechanism has a greater biological complexity, which can not be represented by the Michaelis–Menten and Hill mechanisms, since these models assume an equilibrium relation between the free iron and the iron–transporter complex, and the constancy of the amount of transporters present in the membrane during the process. This gives rise to the need to develop new methods that can capture and represent the complexity of a biological system, even without a detailed knowledge of the system.

Genetic programming algorithms have proven to be a successful tool for modeling complex dynamic problems even when there is incomplete information regarding the characteristics of the system, generating models that perform better than classic biochemical models in terms of representing the experimental data and predictive capacity, without overfitting. In fact, in this work, we designed a fitness function aimed specifically at avoiding model overfitting, thereby enhancing the model’s predictive capacity. The stages of parameter fitting and calculating the generalization error proposed using the Jackknife method allowed a model that better represents the experimental data to be obtained with a higher predictive capacity.

The empirical model obtained using the proposed algorithm captures the key characteristics of the biological phenomena observed experimentally in the apical iron absorption fluxes and initial iron uptake rates. In addition, the subsequent analysis of the model improved the model’s capacity for representing the phenomena and allowed elucidating the contribution of each of the terms that compose the model. In particular, the exponential-quadratic term was associated to the change in the iron uptake velocity, resulting from the internalization of the DMT1 transporters. These results lead to new questions related to this matter, for instance, whether the movement of transporters (or the nonlinear components of the system) are only relevant during the first five minutes of the experiment or if their effect endures, producing new oscillations in the fluxes.

The increasing availability of large biological datasets produced by high-throughput equipment stresses the need for better tools for the mathematical modeling of these data, effectively obtaining information and detecting patterns in an automated manner, especially for systems where the phenomenological knowledge is scarce. In this context, methods like the one presented in this paper are fundamental for data analysis and interpretation, and for the elucidation of biological mechanisms and their key components in complex systems.

## Supporting Information

S1 Table Experimental data of apical uptake over time for different iron challenge concentrations. (XLSX)

S2 Table Experimental data of initial rates for different iron challenge concentrations. (XLSX)
